# The sialidase NEU1 directly interacts with the juxtamembranous segment of the cytoplasmic domain of mucin-1 to inhibit downstream PI3K-Akt signaling

**DOI:** 10.1016/j.jbc.2021.101337

**Published:** 2021-10-22

**Authors:** Sang W. Hyun, Akihiro Imamura, Hideharu Ishida, Kurt H. Piepenbrink, Simeon E. Goldblum, Erik P. Lillehoj

**Affiliations:** 1 US Department of Veterans Affairs, Veterans Affairs Medical Center, University of Maryland School of Medicine, Baltimore, Maryland, USA; 2 Department of Medicine, University of Maryland School of Medicine, Baltimore, Maryland, USA; 3 Department of Applied Bio-organic Chemistry, Gifu University, Gifu, Japan; 4 Food Science and Technology Department, University of Nebraska, Lincoln, Nebraska, USA; 5 Department of Pediatrics, University of Maryland School of Medicine, Baltimore, Maryland, USA

**Keywords:** mucin 1, cell surface associated (MUC1), neuraminidase-1, sialidase, sialic acid, phosphatidylinositide 3-kinase (PI 3-kinase), Akt PBK, Ad, adenovirus, Ad-NEU1-FLAG, adenovirus encoding FLAG-tagged NEU1, CD, cytoplasmic domain, ED, extracellular domain, EGFR, epidermal growth factor receptor, HEK, human embryonic kidney, HRP, horseradish peroxidase, MUC1, mucin 1, NEU, neuraminidase, Ni-NTA, nickel-nitrilotriacetic acid, Pa, *Pseudomonas aeruginosa*, pAkt, phosphorylated Akt, PDGFRβ, platelet-derived growth factor receptor β, PNA, peanut agglutinin, PPCA, protective protein/cathepsin A, PVDF, polyvinylidene fluoride, TLR, toll-like receptor

## Abstract

The extracellular domain (ED) of the membrane-spanning sialoglycoprotein, mucin-1 (MUC1), is an *in vivo* substrate for the lysosomal sialidase, neuraminidase-1 (NEU1). Engagement of the MUC1-ED by its cognate ligand, *Pseudomonas aeruginosa*-expressed flagellin, increases NEU1-MUC1 association and NEU1-mediated MUC1-ED desialylation to unmask cryptic binding sites for its ligand. However, the mechanism(s) through which intracellular NEU1 might physically interact with its surface-expressed MUC1-ED substrate are unclear. Using reciprocal coimmunoprecipitation and *in vitro* binding assays in a human airway epithelial cell system, we show here that NEU1 associates with the MUC1-cytoplasmic domain (CD) but not with the MUC1-ED. Prior pharmacologic inhibition of the NEU1 catalytic activity using the NEU1-selective sialidase inhibitor, C9-butyl amide-2-deoxy-2,3-dehydro-N-acetylneuraminic acid, did not diminish NEU1-MUC1-CD association. In addition, glutathione-S-transferase (GST) pull-down assays using the deletion mutants of the MUC1-CD mapped the NEU1-binding site to the membrane-proximal 36 aa of the MUC1-CD. In a cell-free system, we found that the purified NEU1 interacted with the immobilized GST-MUC1-CD and the purified MUC1-CD associated with the immobilized 6XHis-NEU1, indicating that the NEU1-MUC1-CD interaction was direct and independent of its chaperone protein, protective protein/cathepsin A. However, the NEU1-MUC1-CD interaction was not required for the NEU1-mediated MUC1-ED desialylation. Finally, we demonstrated that overexpression of either WT NEU1 or a catalytically dead NEU1 G68V mutant diminished the association of the established MUC1-CD binding partner, PI3K, to MUC1-CD and reduced downstream Akt kinase phosphorylation. These results indicate that NEU1 associates with the juxtamembranous region of the MUC1-CD to inhibit PI3K-Akt signaling independent of NEU1 catalytic activity.

Glycoconjugates expressed on the surface of all eukaryotic cells contain oligosaccharide chains that terminate with N-acetylneuraminic acid (sialic acid) (1, 2). Because of their terminal location and negative charge, sialic acid residues are strategically positioned to influence intermolecular and cell–cell interactions through steric hindrance and/or electrostatic repulsion. The sialylation state of glycoproteins and glycolipids is dynamically and coordinately regulated through the opposing catalytic activities of sialyltransferases and sialidase/neuraminidase (NEU) (3, 4). NEUs hydrolyze the glycosidic linkage between terminal sialic acid and the adjacent subterminal sugar within glycan chains. Four mammalian NEUs have been identified, NEU1, 2, 3, and 4 (5, 6, 7, 8, 9). Although it lacks the conserved protein structural domains characteristic of canonical NEUs, Klotho, a protein originally associated with aging and senescence, has been reported to exhibit sialidase activity (10).

We previously established NEU1 as the predominant sialidase in human airway epithelial cells (11). In these same cells, *in vitro* (11, 12), and in both murine and human lungs, *in vivo* (13), we have identified the highly sialylated mucin-1 (MUC1) extracellular domain (ED) (MUC1-ED) as an *in vivo* substrate for NEU1. MUC1 comprised an NH_2_-terminal MUC1-ED, a single pass transmembrane domain, and a COOH-terminal MUC1 cytoplasmic domain (CD) (MUC1-CD) (14, 15). The 72-aa MUC1-CD contains binding sites for multiple membrane-bound receptors and cytosolic signaling proteins, including PI3K (16), platelet-derived growth factor receptor β (PDGFRβ) (17), p53 (18, 19), c-Met (18), c-Src (20, 21), epidermal growth factor receptor (EGFR) (22), and β- and γ-catenin (20, 21, 23). The MUC1-ED interacts with the key proteins involved in the establishment and progression of cancer, including intercellular adhesion molecule 1 (24, 25), E-selectin (25), galectin-3 (26), and sialic acid-binding immunoglobulin-type lectin 9 (27). The MUC1-ED also recognizes and responds to *Pseudomonas aeruginosa* (Pa)-expressed flagellin, the major structural protein of the bacterial flagellar filament (28). In the respiratory tract, Pa is one of the most frequent and deadly causes of ventilator-associated pneumonia and participates in the pathogenesis of cystic fibrosis, bronchiectasis, and chronic obstructive pulmonary disease (29). Engagement of the Pa-expressed flagellin with the MUC1-ED rapidly increases MUC1 association with both NEU1 and its chaperone/transport protein, protective protein/cathepsin A (PPCA) (11, 12, 13). Once recruited to MUC1, NEU1 desialylates the MUC1-ED, unmasking cryptic binding sites for Pa flagellin, thereby increasing the binding affinity of flagellin to the MUC1-ED. Furthermore, NEU1-mediated MUC1-ED desialylation unmasks a juxtamembranous Gly-Ser protease recognition site, permitting its proteolysis and release of the MUC1-ED from the cell surface into the bronchoalveolar compartment (12, 13). These combined changes generate a shed, hyperadhesive, flagellin-targeting MUC1-ED decoy receptor that disrupts flagellin-driven processes, including Pa motility, biofilm production, and Pa adhesion to the cell-associated MUC1-ED, and protects against lethal Pa lung infection (13). Although NEU1 was initially described as a lysosomal enzyme (6), sialic acid residues on its substrate, the MUC1-ED, are located on the cell surface (14, 15). The mechanism(s) through which intracellular NEU1 might physically interact with the sialic acid residues within the extracellular MUC1-ED substrate is unknown. The current investigation was undertaken to elucidate the molecular mechanism(s) through which NEU1 physically associates with MUC1, ultimately leading to Pa flagellin-provoked generation of the shed, hyperadhesive, flagellin-targeting MUC1-ED decoy receptor.

## Results

### NEU1 coimmunoprecipitates the MUC1-CD but not the MUC1-ED

**Figure 1:**
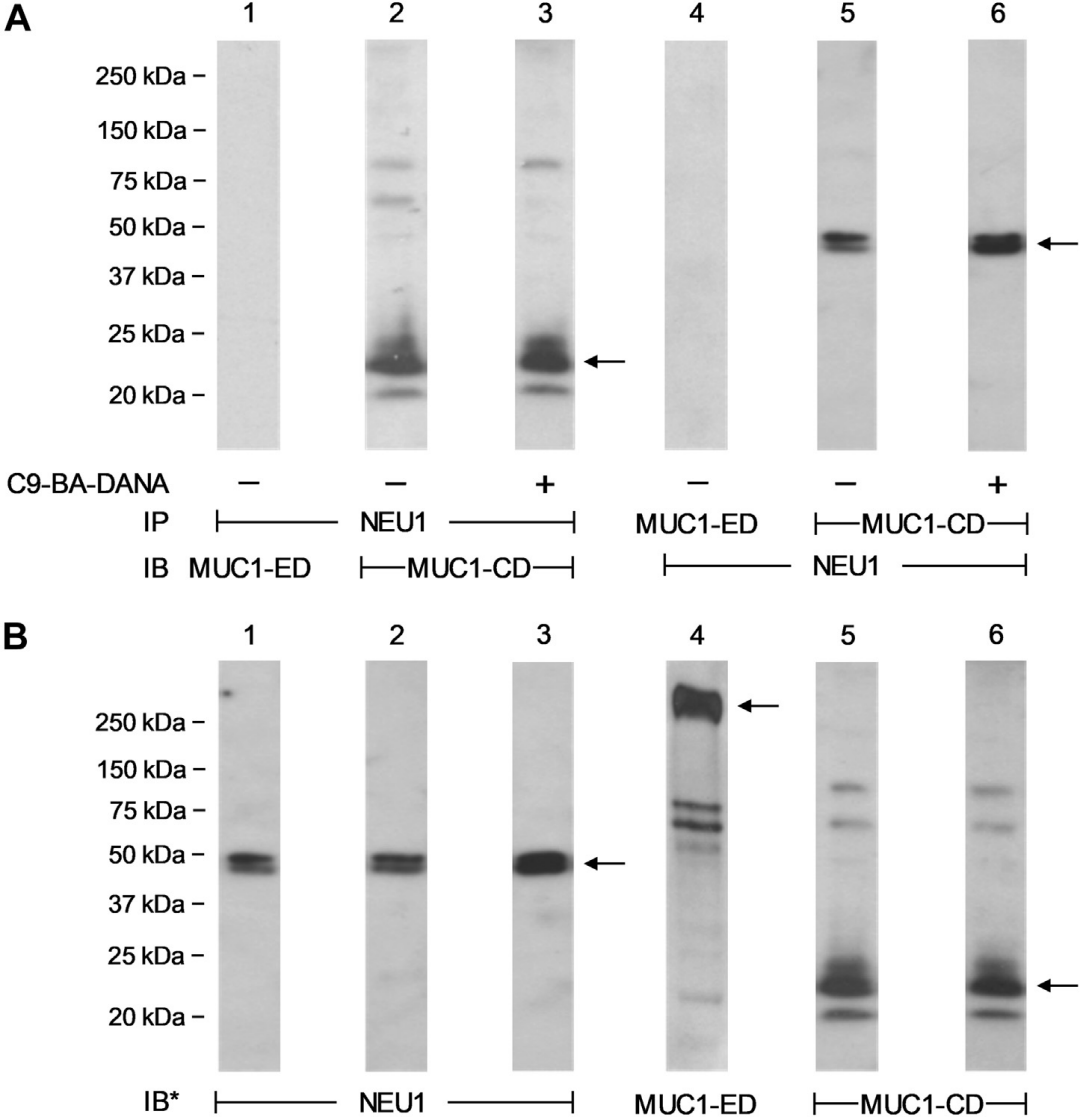
**NEU1 coimmunoprecipitates the MUC1-CD, but not the MUC1-ED.***A* and *B*, the cultured A549 cells were stimulated with Pa-expressed flagellin in the absence (*lanes 1*, *2*, *4*, and *5*) or presence (*lanes 3* and *6*) of the NEU1-selective sialidase inhibitor, C9-butyl amide-2-deoxy-2,3-dehydro-N-acetylneuraminic acid, and lysed in PBS containing 0.5% Triton X-100. The lysates were treated with 0.1% SDS to separate the noncovalently associated MUC1-ED and MUC1-CD subunits and diluted 10-fold in PBS containing 1.0%Triton X-100. *A*, the lysates were immunoprecipitated with anti-NEU1 (*lanes 1–3*), anti-MUC1-ED (*lane 4*), or anti-MUC1-CD (*lanes 5* and *6*) antibodies. The NEU1 immunoprecipitates were processed for MUC1-ED (*lane 1*) or MUC1-CD (*lanes 2* and *3*) immunoblotting, and the MUC1-ED (*lane 4*) and MUC1-CD (*lanes 5* and *6*) immunoprecipitates were processed for NEU1 immunoblotting. The *arrows* indicate the positions of the coimmunoprecipitated MUC1-CD (*lanes 2* and *3*) and NEU1 (*lanes 5* and *6*) protein bands. *B*, to control for protein loading and transfer, the immunoblots were stripped and reprobed with the immunoprecipitating antibody. The molecular weights in kDa are indicated on the *left*. The results are representative of two independent experiments. IB, immunoblot; IB∗, immunoblot after stripping; IP, immunoprecipitation; MUC1-CD, mucin-1 cytoplasmic domain; MUC1-ED, mucin-1 extracellular domain; NEU1, neuraminidase-1; Pa, *Pseudomonas aeruginosa*.

We previously demonstrated that the engagement of the MUC1-ED with its cognate ligand, Pa-expressed flagellin, rapidly increases NEU1 association with MUC1 followed by desialylation of the MUC1-ED substrate (11, 12, 13). For intracellular NEU1 to gain access to the surface-expressed, sialylated MUC1-ED substrate, we asked to which portion of MUC1 might NEU1 bind. Here, A549 cells were stimulated with Pa-derived flagellin, lysed, and the lysates were processed for reciprocal NEU1/MUC1-ED and NEU1/MUC1-CD coimmunoprecipitation assays. NEU1 immunoprecipitation coimmunoprecipitated MUC1-CD (Fig. 1*A*, lane 2), but not MUC1-ED (Fig. 1*A*, lane 1). Similarly, MUC1-CD immunoprecipitation coimmunoprecipitated NEU1 (Fig. 1*A*, lane 5), whereas MUC1-ED immunoprecipitation did not (Fig. 1*A*, lane 4). Prior NEU1-selective sialidase inhibition with C9-butyl amide-2-deoxy-2,3-dehydro-N-acetylneuraminic acid failed to diminish the NEU1 coimmunoprecipitation of MUC1-CD (Fig. 1*A*, lanes 3 *versus* 2) or MUC1-CD coimmunoprecipitation of NEU1 (Fig. 1*A*, lanes 6 *versus* 5). As an another measure to exclude NEU1–MUC1-ED association, cell-free bronchoalveolar lavage fluid harvested from patients with Pa lung infections that contain the shed MUC1-ED, but not the membrane-tethered MUC1-CD (12), were processed for reciprocal NEU1/MUC1-ED coimmunoprecipitation assays. Immunoprecipitation of NEU1 failed to coimmunoprecipitate the MUC1-ED (Fig. S1*A*, lanes 1–4), and immunoprecipitation of the MUC1-ED failed to coimmunoprecipitate NEU1 (Fig. S1*A*, lanes 5–8). These combined data indicate that NEU1 directly/indirectly associates with the MUC1-CD, but not the MUC1-ED, and that NEU1 catalytic activity is not required for the NEU1-MUC1-CD interaction.

### NEU1 associates with the MUC1-CD but not the MUC1s-ED in *in vitro* binding assays

**Figure 2:**
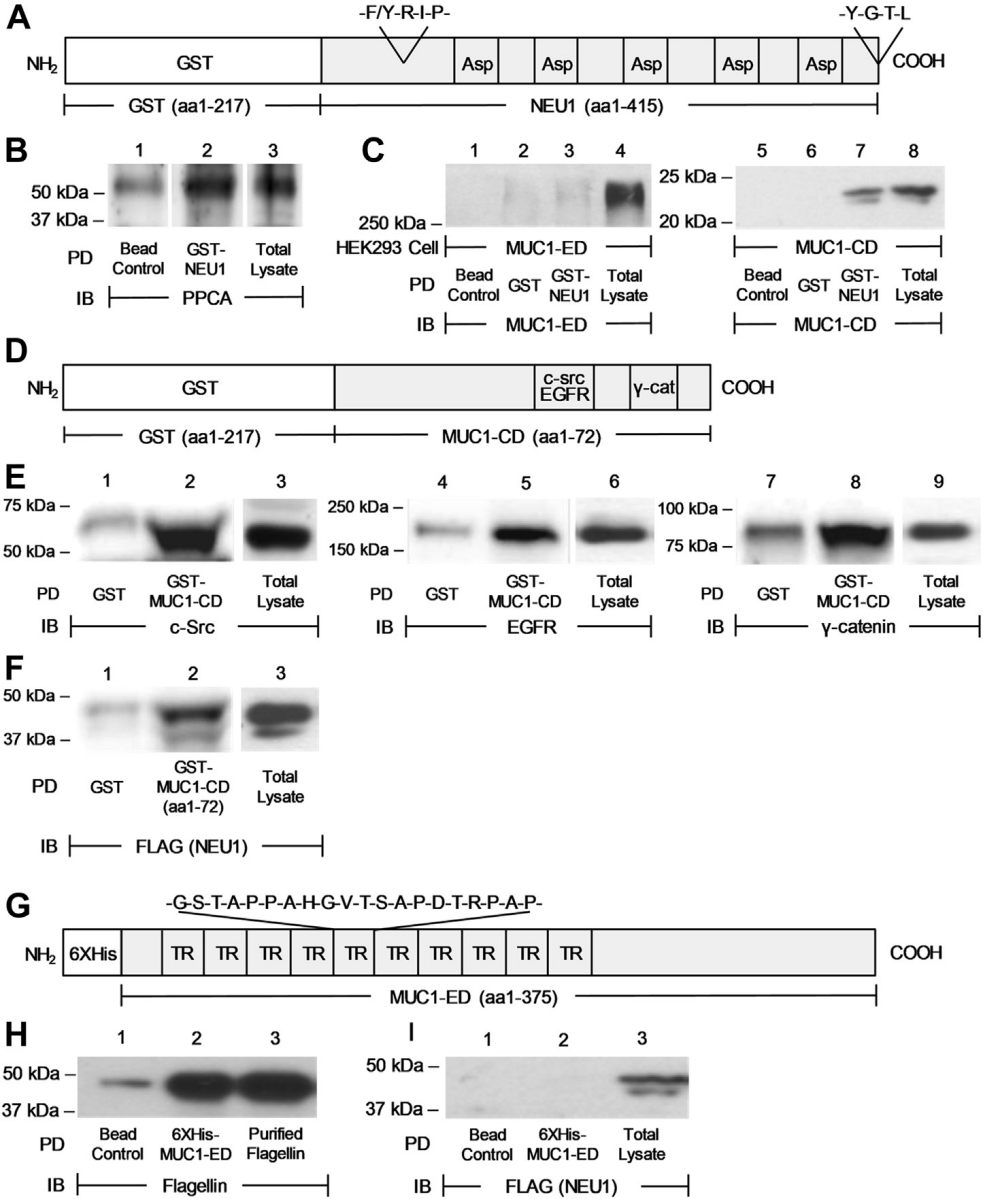
**NEU1 associates with the MUC1-CD, but not the MUC1-ED, in *in vitro* binding assays.***A*, schematic of the glutathione-S-transferase (GST)-NEU1 recombinant protein containing a 217-aa GST protein fused to the NH_2_-terminus of the 415-aa full-length NEU1 protein. In the NEU1 schematic, 5 Asp boxes, a conserved -F/Y-R-I-P- sequence, and a COOH-terminal Tyr phosphorylation motif (-Y-G-T-L) are indicated. *B*, A549 cells were lysed and the lysates incubated with glutathione-agarose beads alone (*lane 1*) or GST-NEU1 immobilized on glutathione-agarose beads (*lane 2*), or were directly loaded on the gel (*lane 3*). Proteins bound to the beads and the total lysates were processed for PPCA immunoblotting. *C*, HEK293T cells were transfected for MUC1-ED (*lanes 1–4*) or MUC1-CD (*lanes 5–8*) expression, cultured for 48 h, and lysed. The lysates were incubated with beads alone (*lanes 1* and *5*), GST (*lanes 2* and *6*), or the validated GST-NEU1 (*lanes 3* and *7*), each immobilized on the beads, or were directly loaded on the gel (*lanes 4* and *8*). Proteins bound to the beads and the total lysates were processed for MUC1-ED (*lanes 1–4*) or MUC1-CD (*lanes 5–8*) immunoblotting. *D*, schematic of the GST-MUC1-CD (aa1–72) recombinant protein. The binding sites for c-Src, EGFR, and γ-catenin in the MUC1-CD are indicated. *E*, A549 cell lysates were incubated with GST or GST-MUC1-CD (aa1–72), each immobilized on glutathione-agarose beads, or were directly loaded on the gel. Proteins bound to the beads and the total lysates were processed for c-Src (*lanes 1–3*), EGFR (*lanes 4–6*), or γ-catenin (*lanes 7–9*) immunoblotting. *F*, A549 cells were infected with Ad-NEU1-FLAG at a multiplicity of infection (m.o.i.) = 100, cultured for 48 h, and lysed. The lysates were incubated with GST or the validated GST-MUC1-CD (aa1–72), each immobilized on glutathione-agarose beads, or were directly loaded on the gel. Proteins bound to the beads and the total lysates were processed for FLAG (NEU1) immunoblotting. *G*, schematic of the 6XHis-MUC1-ED recombinant protein containing a 6-aa His epitope tag fused to the NH_2_-terminus of the 375-aa MUC1-ED protein. The tandem repeat (TR)s (G-S-T-A-P-P-A-H-G-V-T-S-A-P-D-T-R-P-A-P) within the MUC1-ED are indicated. *H*, the purified Pa-derived flagellin was incubated with Ni-NTA-agarose beads alone (*lane 1*) or 6XHis-MUC1-ED immobilized on Ni-NTA-beads (*lane 2*), or was directly loaded on the gel (*lane 3*). Proteins bound to the beads and the loaded flagellin gel mobility control were processed for flagellin immunoblotting. *I*, A549 cells were infected with Ad-NEU1-FLAG (m.o.i. = 100), incubated for 48 h, and lysed. The lysates were incubated with Ni-NTA-agarose beads alone (*lane 1*) or the validated 6XHis-MUC1-ED immobilized on Ni-NTA-beads (*lane 2*), or were directly loaded on the gel (*lane 3*). Proteins bound to the beads and the total lysates were processed for FLAG (NEU1) immunoblotting. The results are representative of 3 independent experiments. *B–F*, *H*, and *I*, the molecular weights in kDa are indicated on the *left*. Ad-NEU1-FLAG, adenovirus encoding FLAG-tagged NEU1; EGFR, epidermal growth factor receptor; HEK, human embryonic kidney; IB, immunoblot; MUC1-CD, mucin-1 cytoplasmic domain; MUC1-ED, mucin-1 extracellular domain; NEU1, neuraminidase-1; Ni-NTA, nickel-nitrilotriacetic acid; PD, pull-down; PPCA, protective protein/cathepsin A.

To confirm that NEU1 associates with the MUC1-CD, but not the MUC1-ED, *in vitro* binding assays were performed with glutathione-S-transferase (GST) NEU1 immobilized on glutathione-agarose. The GST-NEU1 recombinant protein encompassed the 217-aa *Schistosoma japonicum* GST protein fused to the NH_2_-terminus of the 415-aa full-length NEU1 protein and included its highly conserved Phe/Tyr-Arg-Ile-Pro motif and 5 Asp boxes (Fig. 2*A*). To validate the GST-NEU1 construct, we found that GST-NEU1 immobilized on glutathione-agarose bound to the established NEU1-binding partner, PPCA, to a much greater extent than did glutathione-agarose alone (Fig. 2*B*, lane 2 *versus* lane 1). Human embryonic kidney (HEK)293T cells transfected for the expression of either the MUC1-ED or the MUC1-CD were lysed. The lysates were incubated with the immobilized GST-NEU1 and the NEU1-binding proteins processed for MUC1-ED or MUC1-CD immunoblotting. GST-NEU1 associated with the MUC1-CD (Fig. 2*C*, lane 7 *versus* 6), but not with the MUC1-ED (Fig. 2*C*, lane 3 *versus* 2).

To further establish the NEU1–MUC1-CD interaction, we constructed a GST-fusion protein encompassing the entire 72-aa MUC1-CD (aa1–72) (Fig. 2*D*). To validate the GST–MUC1-CD (aa1–72) construct, we found that in GST pull-down assays, it associated with 3 established MUC1-CD binding partners, c-Src (30) (Fig. 2*E*, lane 2 *versus* 1), EGFR (22) (Fig. 2*E*, lane 5 *versus* 4), and γ-catenin (31) (Fig. 2*E*, lane 8 *versus* 7). We then demonstrated that the validated GST-MUC1-CD (aa1–72) binds to NEU1 (Fig. 2*F*, lane 2 *versus* 1). To further support that NEU1 did not associate with the MUC1-ED, binding assays were performed using the MUC1-ED protein containing an NH_2_-terminal 6XHis epitope tag (Fig. 2*G*) immobilized on nickel-nitrilotriacetic acid (Ni-NTA)-agarose. To validate the 6XHis-MUC1-ED construct, in Ni-NTA-agarose pull-down assays, MUC1-ED associated with its established ligand, Pa-expressed flagellin, dramatically more than did the Ni-NTA-agarose bead control (Fig. 2*H*, lane 2 *versus* lane 1). The lysates of human airway cells infected with recombinant adenovirus encoding FLAG-tagged NEU1 (Ad-NEU1-FLAG) were similarly incubated with the validated 6XHis-MUC1-ED immobilized on Ni-NTA-agarose or Ni-NTA-agarose alone and the MUC1-ED binding proteins processed for FLAG (NEU1) immunoblotting. NEU1 binding to the immobilized MUC1-ED was not detected (Fig. 2*I*, lane 2). Therefore, in a tightly controlled *in vitro* system, again, NEU1 directly/indirectly interacts with the MUC1-CD but not with the MUC1-ED.

### NEU1 fails to bind to the MUC1-ED deletion mutants

We next asked whether the inability of NEU1 to bind to the MUC1-ED might be explained through intramolecular interference of NEU1 binding to one or more site(s) within the MUC1-ED by other portions of the same MUC1-ED molecule. Six 6XHis epitope-tagged deletion mutants (6XHis-MUC1-ED-Δ1, -Δ2, -Δ3, -Δ4, -Δ5, and -Δ6) were constructed (Fig. S2*A*). Lysates of A549 cells infected with Ad-NEU1-FLAG were incubated with the 6XHis-MUC1-ED mutant constructs immobilized on Ni-NTA-agarose, and the MUC1-ED bound proteins processed for FLAG (NEU1) immunoblotting (Fig. S2*B*, lanes 1–7). None of the immobilized MUC1-ED mutant proteins exhibited NEU1 binding. As a loading and transfer control, A549 cell proteins that were not retained by the immobilized MUC1-ED-WT or its deletion mutants were similarly processed for FLAG (NEU1) immunoblotting, revealing NEU1 immunoreactive bands at the expected gel mobility corresponding to 45.5 kDa (11) (Fig. S2*C*, lanes 1–8). In the reciprocal approach, the MUC1-ED-WT and MUC1-ED mutant proteins expressed in the HEK293T cells were each adsorbed to Ni-NTA-agarose beads and eluted with imidazole. The purified, unbound MUC1-ED-WT and deletion mutants were incubated with the validated GST-NEU1 immobilized on glutathione-agarose beads, and the NEU1-binding proteins processed for 6XHis (MUC1-ED) immunoblotting (Fig. S2*D*, lanes 1–4, 6–8). Again, MUC1-ED binding to the immobilized NEU1 was not detected. As a positive control, MUC1-CD expressed in the HEK293T cells associated with the immobilized GST-NEU1 (Fig. S2*D*, lanes 5, 9). As an added control, the MUC1-ED-WT and mutant proteins that did not bind to the immobilized GST-NEU1 were processed for 6XHis (MUC1-ED) immunoblotting. Here, immunostaining revealed protein bands with the anticipated gel mobilities of the respective MUC1-ED-WT and mutant proteins (Fig. S2*E*, lanes 1–7). These combined results suggest that intramolecular interference of any NEU1-MUC1-ED interaction is unlikely.

### NEU1 binds to the juxtamembranous region of the MUC1-CD

**Figure 3:**
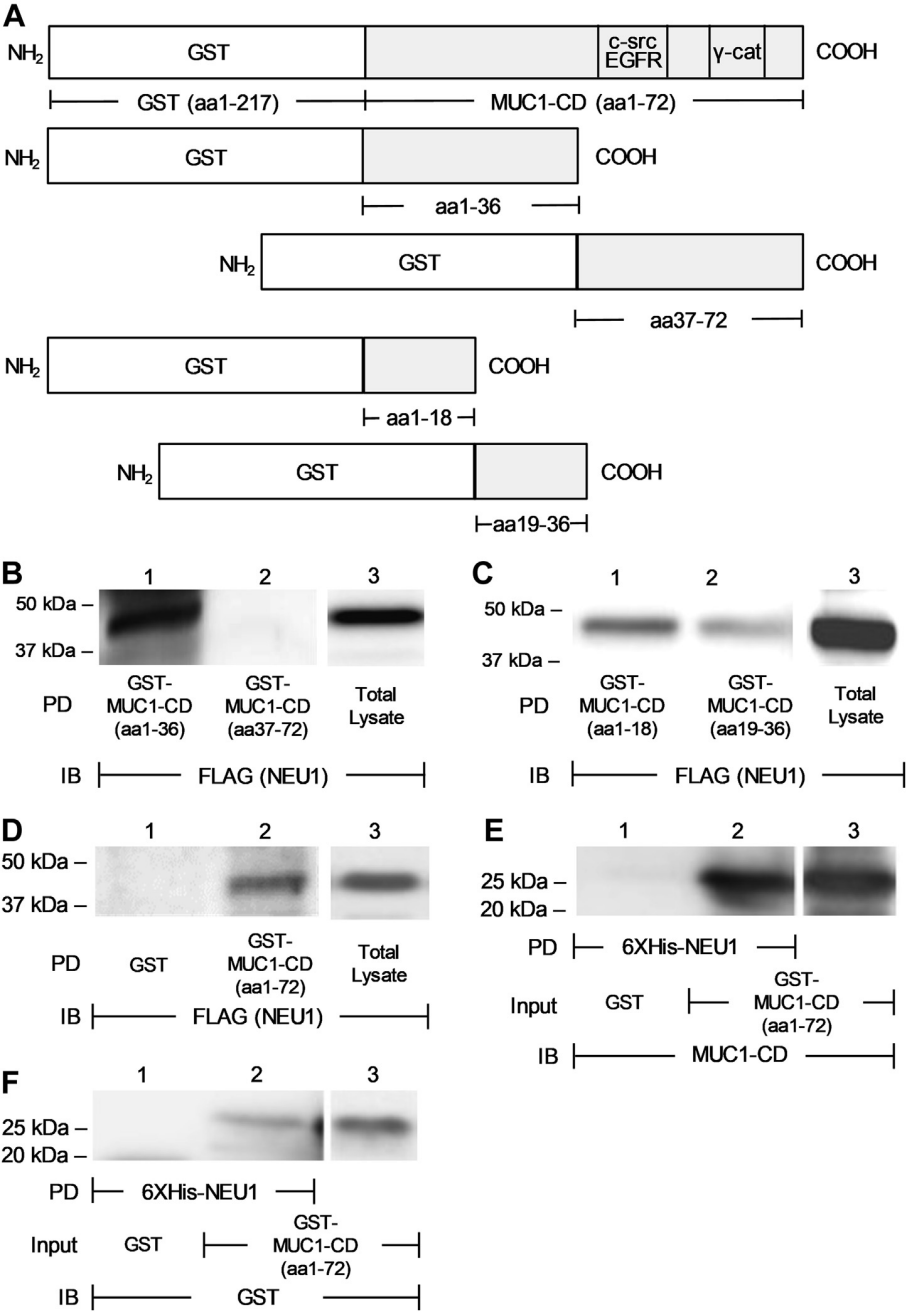
**NEU1 associates with the juxtamembranous region of the MUC1-CD.***A*, schematic of the glutathione-S-transferase (GST)-MUC1-CD (aa1–72), GST-MUC1-CD (aa1–36), GST-MUC1-CD (aa37–72), GST-MUC1-CD (aa1–18), and GST-MUC1-CD (aa19–36) recombinant proteins. *B*, A549 cells were infected with Ad-NEU1-FLAG (m.o.i. = 100), cultured for 48 h, and lysed. The lysates were incubated with GST-MUC1-CD (aa1–36) or GST-MUC1-CD (aa37–72), each immobilized on glutathione-agarose beads, or were directly loaded on the gel. *C*, the lysates were incubated with GST-MUC1-CD (aa1–18) or GST-MUC1-CD (aa19–36), each immobilized on glutathione-agarose beads, or were directly loaded on the gel. *D*, the lysates were incubated with GST or GST-MUC1-CD (aa1–72) each immobilized on glutathione-agarose beads, after which the beads were incubated with Factor Xa to proteolytically release the bound proteins, and the released proteins purified on a GST trap column. *B–F*, the proteins bound to beads, eluted proteins, and total lysates were all processed for FLAG (NEU1) immunoblotting. *E* and *F*, GST (*lane 1*) and GST-MUC1-CD (*lane 2*), each immobilized on glutathione-agarose beads, were eluted with free glutathione and incubated with 6XHis-NEU1 coupled to Ni-NTA-agarose beads. The 6XHis-NEU1-binding proteins and purified GST-MUC1-CD (*lane 3*) were processed for (*E*) MUC1-CD or (*F*) GST immunoblotting. The results are representative of two to three independent experiments. *B–F*, The molecular weights in kDa are indicated on the *left*. Ad-NEU1-FLAG, adenovirus encoding FLAG-tagged NEU1; EGFR, epidermal growth factor receptor; IB, immunoblot; MUC1-CD, mucin-1 cytoplasmic domain; NEU1, neuraminidase-1; Ni-NTA, nickel-nitrilotriacetic acid; PD, pull-down.

To begin to define the MUC1-CD structural requirements for its association with NEU1, we constructed GST fusion proteins comprising its 36-aa membrane proximal NH_2_-terminal half (aa1–36) and its 36-aa COOH-terminal half (aa37–72) (Fig. 3*A*). We found that NEU1 associated with GST-MUC1-CD (aa1–36) (Fig. 3*B*, lane 1), but not with GST-MUC1-CD (aa37–72) (Fig. 3*B*, lane 2), mapping the NEU1 binding site to the NH_2_-terminal juxtamembrane half of the MUC1-CD. Additional binding assays using GST-MUC1-CD (aa1–18) and GST-MUC1-CD (aa19–36) (Fig. 3*A*) revealed preferential NEU1 association with the MUC1-CD (aa1–18) with lesser association with the MUC1-CD (aa19–36) (Fig. 3*C*, lanes 1, 2). These combined data indicate that NEU1 binds to the juxtamembranous region of the MUC1-CD.

### NEU1 binds directly to the MUC1-CD

To determine whether the NEU1–MUC1-CD interaction was direct, binding assays were performed using NEU1 that had been proteolytically cleaved from its GST epitope tag by Factor Xa and purified on a GST trap column. The purified NEU1 associated with GST-MUC1-CD (aa1–72) immobilized on glutathione-agarose, but not with immobilized GST alone (Fig. 3*D*, lane 2 *versus* 1). In the reciprocal approach, 6XHis-NEU1 was immobilized on Ni-NTA-agarose and incubated with either GST-MUC1-CD (aa1–72) or GST alone, each of which was eluted from glutathione-agarose with free glutathione. The purified GST-MUC1-CD (aa1–72) associated with immobilized 6XHis-NEU1, whereas GST alone did not, as revealed by both MUC1-CD (Fig. 3*E*, lane 2 *versus* 1) and GST (Fig. 3*F*, lane 2 *versus* 1) immunoblotting. Taken together, these results indicate that NEU1 specifically and directly interacts with the juxtamembranous portion of the MUC1-CD.

### PPCA and MUC1-CD each bind to the NH_2_-terminal portion of NEU1

**Figure 4:**
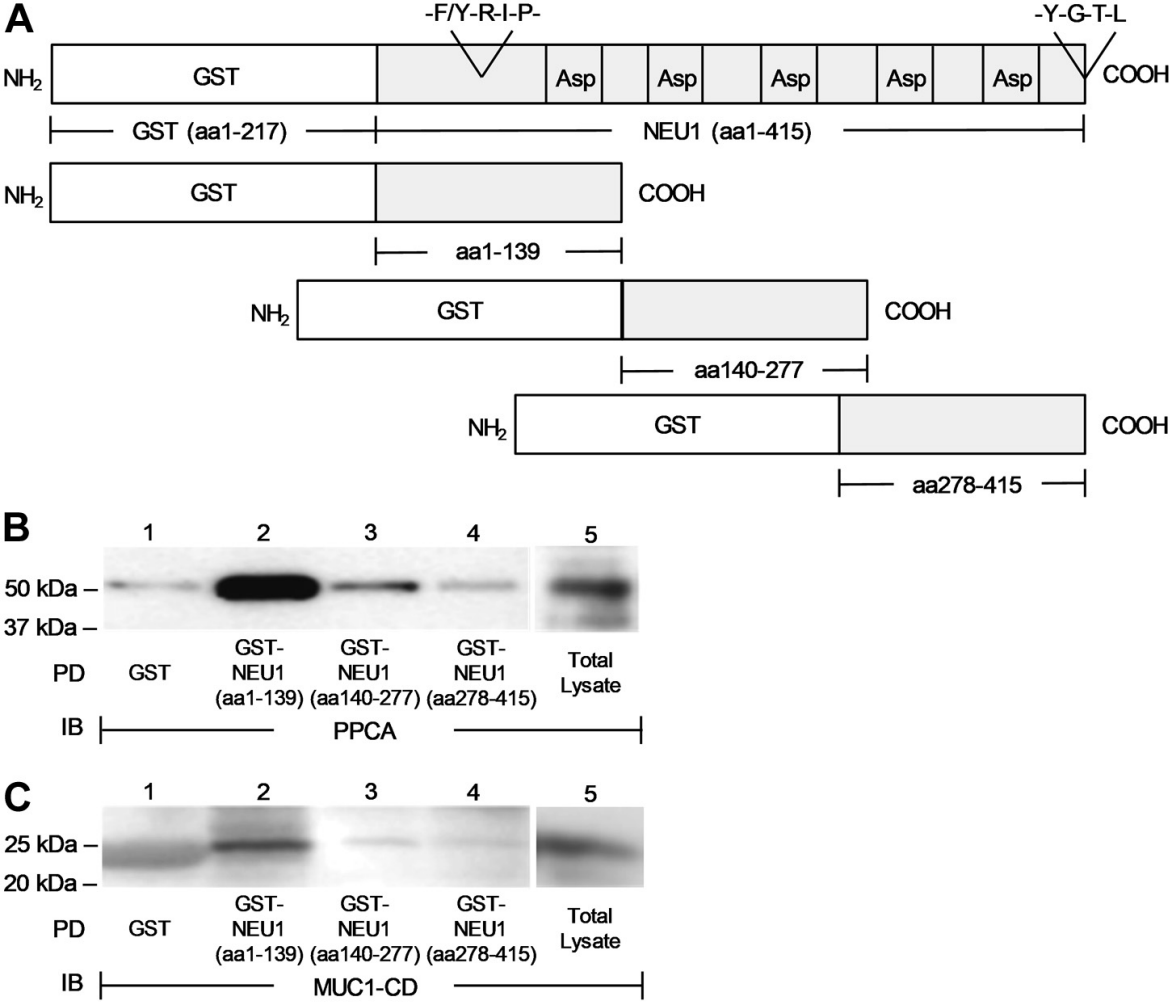
**PPCA and MUC1-CD each bind to the NH**_**2**_**-terminal portion of NEU1.***A*, schematic of glutathione-S-transferase (GST)-NEU1 (aa1–415), GST-NEU1 (aa1–139), GST-NEU1 (aa140–277), and GST-NEU1 (aa278–415) recombinant proteins. *B* and *C*, A549 cell lysates were incubated with GST, GST-NEU1 (aa1–139), GST-NEU1 (aa140–277), or GST-NEU1 (aa278–415), each immobilized on glutathione-agarose beads, or were directly loaded on the gel. Proteins bound to the beads and the total lysates were processed for (*B*) PPCA or (*C*) MUC1-CD immunoblotting. The results are representative of two independent experiments. The molecular weights in kDa are indicated on the *left*. IB, immunoblot; MUC1-CD, mucin-1 cytoplasmic domain; NEU1, neuraminidase-1; PD, pull-down; PPCA, protective protein/cathepsin A.

To identify the region(s) of NEU1 that associate(s) with the MUC1-CD and PPCA, 3 GST-tagged NEU1 deletion mutants were constructed encompassing NEU1 (aa1–139), NEU1 (aa140–277), and NEU1 (aa278–415) (Fig. 4*A*). The lysates of A549 cells were incubated with GST-NEU1 (aa1–139), GST-NEU1 (aa140–277), or GST-NEU1 (aa278–415), each immobilized on glutathione-agarose, and the NEU1-binding proteins processed for PPCA (Fig. 4*B*) or MUC1-CD (Fig. 4*C*) immunoblotting. PPCA (Fig. 4*B*, lanes 2 *versus* 3 and 4) and the MUC1-CD (Fig. 4*C*, lanes 2 *versus* 3 and 4) each associated with GST-NEU1 (aa1–139) to a much greater extent than it associated with GST-NEU1 (aa140–277) or GST-NEU1 (aa278–415). Therefore, both the MUC1-CD and PPCA, each bind to the NH_2_-terminal segment of NEU1.

### The MUC1-CD is not required for Pa flagellin-stimulated, NEU1-mediated MUC1-ED desialylation

**Figure 5:**
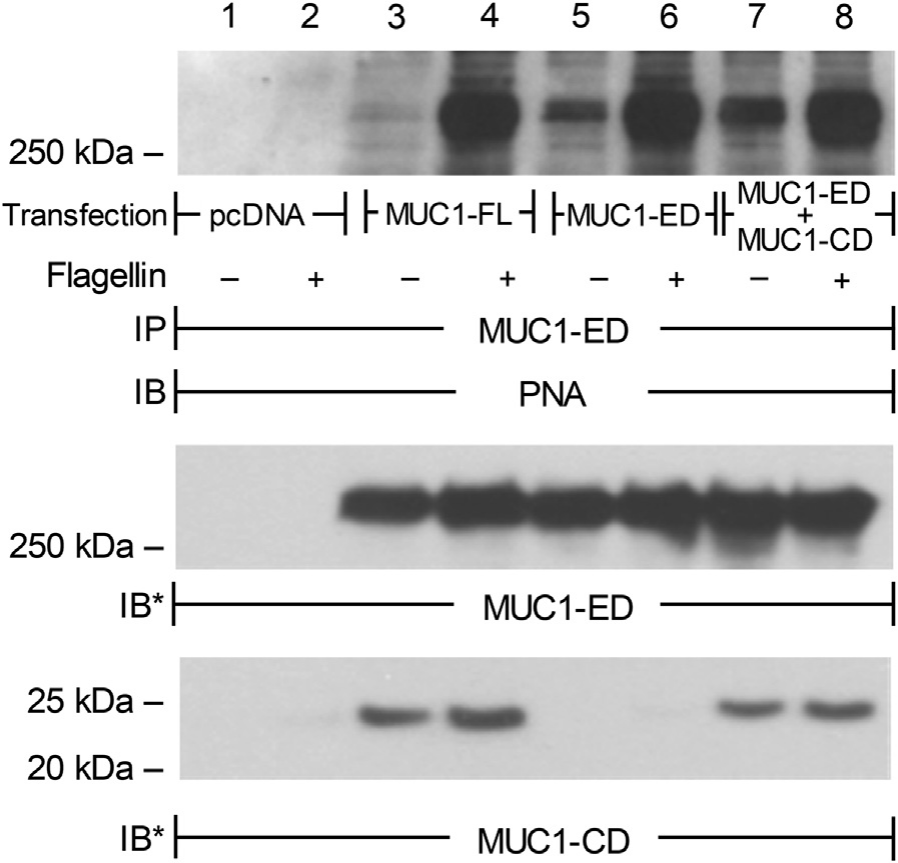
**The MUC1-CD is not required for Pa-flagellin-stimulated, NEU1-mediated MUC1-ED desialylation.** HEK293T cells were transfected with the empty pcDNA plasmid (*lanes 1* and *2*) or with plasmids encoding the full-length (FL) MUC1 (*lanes 3* and *4*), MUC1-ED (*lanes 5* and *6*), or MUC1-ED plus MUC1-CD (*lanes* 7 and *8*) and cultured for 48 h. The cells were incubated for 30 min with 10 ng/ml of Pa-expressed flagellin or medium alone and lysed. The lysates were immunoprecipitated with anti-MUC1-ED antibody and the immunoprecipitates processed for PNA lectin blotting (*upper panel*). To control for loading and transfer, the blots were stripped and reprobed for MUC1-ED (*middle panel*) and MUC1-CD (*lower panel*). The results are representative of three independent experiments. The molecular weights in kDa are indicated on the *left*. HEK, human embryonic kidney; IB, immunoblot; IB∗, immunoblot after stripping; IP, immunoprecipitation; MUC1-CD, mucin-1 cytoplasmic domain; MUC1-ED, mucin-1 extracellular domain; MUC1-FL, mucin-1 full-length; NEU1, neuraminidase-1; PNA, peanut agglutinin.

We asked whether NEU1 association with the MUC1-CD (Fig. 2*C*, lane 7; Fig. 2*I*, lane 2; Fig. 4*C*, lane 2) was required for NEU1-mediated MUC1-ED desialylation. The HEK293T cells were transfected with the full-length MUC1, MUC1-ED, or MUC1-ED plus MUC1-CD constructs, stimulated with Pa flagellin, and MUC1-ED desialylation assessed by peanut agglutinin (PNA) lectin blotting. We previously showed that use of the galactose-binding lectin, PNA, to detect increased NEU1-mediated MUC1-ED desialylation was more sensitive than were the sialic acid-binding lectins, *Maackia amurensis* lectin II or *Sambucus nigra* agglutinin, to detect decreased MUC1-ED sialylation (12). Flagellin dramatically increased MUC1-ED desialylation in the cells transfected with constructs encoding the full-length MUC1, MUC1-ED, or MUC1-ED plus MUC1-CD, compared with unstimulated cells (Fig. 5, lanes 4 *versus* 3, 6 *versus* 5, and 8 *versus* 7). However, no increase in MUC1-ED desialylation was evident in cells expressing the full-length MUC1 or MUC1-ED plus MUC1-CD constructs compared with cells expressing only the MUC1-ED construct (Fig. 5, lanes 4 and 8 *versus* 6). These combined data indicate that the MUC1-CD is not required for flagellin-stimulated, NEU1-mediated MUC1-ED desialylation.

### NEU1 binding to the MUC1-CD disrupts PI3K-Akt signaling

**Figure 6:**
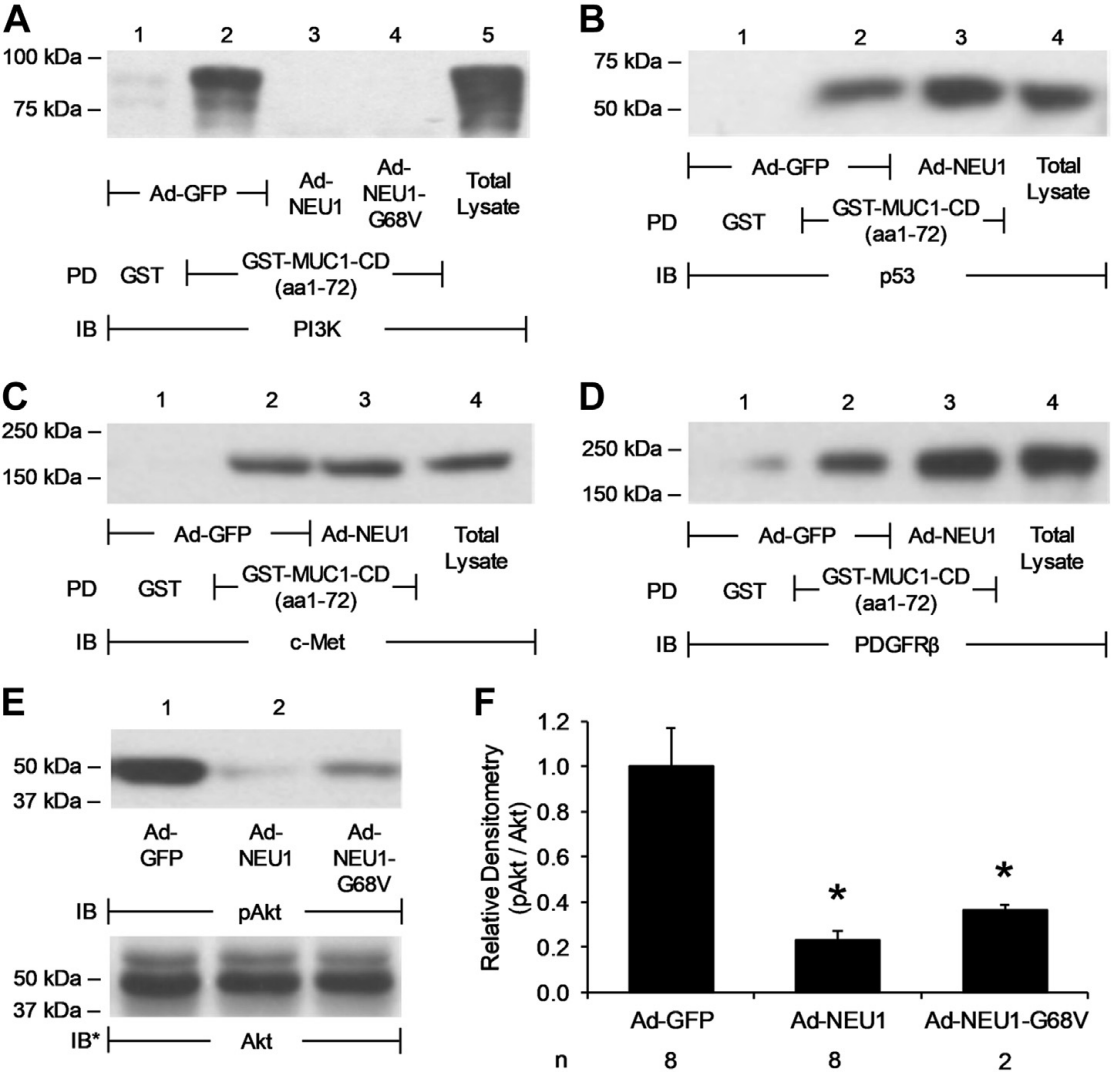
**NEU1 interaction with the MUC1-CD (aa1–72) counter-regulates the PI3K/Akt pathway.***A–E*, A549 cells were infected with Ad-GFP, Ad-NEU1, or Ad-NEU1-G68V (m.o.i. = 100), cultured for 48 h, and lysed. *A*, the lysates were incubated with glutathione-S-transferase (GST) (*lane 1*) or GST-MUC1-CD (aa1–72) (*lanes 2–4*), each immobilized on glutathione-agarose beads, or were directly loaded on the gel (*lane 5*). *B–D*, the lysates were incubated with GST (*lane 1*) or GST-MUC1-CD (aa1–72) (*lanes 2* and *3*), each immobilized on glutathione-agarose beads, or were directly loaded on the gel (*lane 4*). Proteins bound to the beads and the total lysates were processed for (*A*) PI3K, (*B*) p53, (*C*) c-Met, or (*D*) PDGFRβ immunoblotting. *E*, the lysates were processed for pAkt immunoblotting (*upper panel*). To control for protein loading and transfer, the immunoblot was stripped and reprobed for total Akt (*lower panel*). *F*, densitometric analyses of the blots in (*E*). The error bars represent mean ± S.E. pAkt signal normalized to the total Akt signal in the same lane on the same stripped and reprobed blot (n = 2, 6). The results are representative of two to six independent experiments. The molecular weight in kDa is indicated on the *left*. ∗, decreased normalized pAkt signal of Ad-NEU1- and Ad-NEU1-G68V-infected cells each compared with Ad-GFP-infected cells at *p* < 0.05. Ad, adenovirus; IB, immunoblot; IB∗, immunoblot after stripping; MUC1-CD, mucin-1 cytoplasmic domain; NEU1, neuraminidase-1; pAkt, phosphorylated Akt; PD, pull down; PDGFRβ, platelet-derived growth factor receptor β.

Multiple binding partners for the MUC1-CD (aa1–36) region have been identified, including PI3K, p53, c-Met, and PDGFRβ (16, 17, 18, 19). We asked whether NEU1 bound to the MUC1-CD (aa1-36) (Fig. 3*B*, lane 1) might interfere with the binding to one or more of these established MUC1-CD (aa1–36) binding partners. Forced NEU1 overexpression in A549 cells using Ad-NEU1 infection diminished PI3K–MUC1-CD association compared with cells infected with Ad-GFP (Fig. 6*A*, lanes 3 *versus* 2). Furthermore, infection of A549 cells with Ad-NEU1-G68V encoding a catalytically dead NEU1 mutant also diminished PI3K–MUC1-CD association (Fig. 6*A*, lanes 4 *versus* 2), indicating that NEU1 catalytic activity was not required for NEU1-mediated disruption of PI3K–MUC1-CD association. In contrast, infection of A549 cells with Ad-NEU1 did not influence the association of p53 (Fig. 6*B*, lanes 3 *versus* 2), c-Met (Fig. 6*C*, lanes 3 *versus* 2), or PDGFRβ (Fig. 6*C*, lanes 3 *versus* 2) with the MUC1-CD. To determine whether NEU1 might also diminish Akt activation, a signaling event downstream of PI3K (32), lysates of Ad-GFP-, Ad-NEU1-, or Ad-NEU1-G68V-infected A549 cells were processed for phosphorylated (p)Akt immunoblotting. Infection with Ad-NEU1 reduced mean normalized pAkt levels by 73.6%, and infection with Ad-NEU1-G68V reduced mean normalized pAkt levels by 60.4%, each compared with that observed with Ad-GFP-infected cells (Fig. 6*E*, lanes 2, 3 *versus* 1, Fig. 6*F*). These combined results indicate that NEU1 binding to the MUC1-CD disrupts PI3K–Akt signaling independent of its catalytic activity.

## Discussion

NEU1 was originally described as a lysosomal sialidase catalyzing the removal of terminal sialic acids from sialoglycoconjugates (6). However, multiple reports have documented its localization to the cell surface (33, 34, 35, 36, 37, 38, 39, 40, 41, 42, 43, 44, 45), and numerous cell surface sialoglycoproteins have been identified as NEU1 substrates (11, 33, 44, 46, 47, 48, 49, 50, 51, 52, 53, 54, 55, 56, 57). We have established the membrane-spanning MUC1 mucin as an *in vivo* NEU1 substrate (11, 12, 13). MUC1 is a noncovalently-associated complex, which comprised a > 250 kDa, NH_2_-terminal, heavily sialylated MUC1-ED coupled to a relatively short, 72-aa COOH-terminal MUC1-CD (14, 15). The MUC1-ED contains a variable number of 20-aa tandem repeats containing more than 500 potential O-linked glycosylation sites, of which 70 to 90% are terminally sialylated. The MUC1-ED also contains a Gly-Ser protease recognition site located 58 aa upstream of the transmembrane domain. The MUC1-CD contains binding sites for multiple cytosolic signaling molecules (14, 15). To provide insight into how intracellular NEU1 might gain access to extracellular sialylated glycan chains of the MUC1-ED, we asked which molecular components participate in the NEU1-MUC1 interaction. Our key findings include the following: (1) NEU1 binds to the MUC1-CD, but not to the MUC1-ED, (2) NEU1 (aa1–139) binds to the juxtamembranous 36 aa of the MUC1-CD, (3) the NEU1–MUC1-CD interaction is direct, independent of its chaperone, PPCA, (4) the MUC1-CD is not required for NEU1-mediated MUC1-ED desialylation, (5) NEU1 binding to the MUC1-CD diminishes PI3K–MUC1-CD association and downstream Akt phosphorylation, and finally, (6) using two different methods, pharmacologic inhibition and overexpression of an enzymatically inactive mutant, NEU1 catalytic activity is not required for either the NEU1–MUC1-CD interaction or NEU1-mediated disruption of the MUC1-CD–PI3K–Akt pathway. To our knowledge, this is the first report of a noncatalytic signaling function for NEU1 sialidase.

We previously reported that the MUC1-CD associates with both NEU1 and PPCA in reciprocal coimmunoprecipitation assays, using cultured airway epithelial cells, *in vitro* (12), and murine lungs, *in vivo* (13). Furthermore, the NEU1–MUC1-CD and PPCA–MUC1-CD protein–protein interactions were increased after either Pa or flagellin stimulation in cell cultures (12) or intrapulmonary Pa or flagellin challenge in mice (13). The current data indicate that both the MUC1-CD and PPCA associate with the NH_2_-terminal NEU1 (aa1–139) region. Previous studies with human erythrocytes demonstrated NEU1 as a peripheral membrane protein in the absence of PPCA (38). Here, NEU1 was present on the outer leaflet of isolated erythrocyte plasma membranes prepared by hypotonic lysis and released from the erythrocyte ghosts by alkaline treatment. In another study using an *in silico* approach, two putative transmembrane regions of the NEU1 sialidase were identified, NEU1 (aa139–159) and NEU1 (aa316–333) (39). In these same studies, catalytically active NEU1, in the absence of PPCA, was demonstrated on the surface of both COS7 cells ectopically overexpressing NEU1 and human macrophages endogenously expressing NEU1. Immunofluorescence studies with NEU1 recombinant proteins containing NH_2_- or COOH-terminal epitope tags and fluoroprobe-labeled antibodies against the epitope tags suggested that the sialidase was oriented on the cell surface as a double-pass integral membrane protein with its termini oriented toward the intracellular compartment and its central loop region facing the extracellular space. This predicted topology places the NEU1 (aa1–139) region, which binds to both PPCA and MUC1-CD, within the intracellular compartment, immediately proximal to the putative NEU1 (aa139–159) transmembrane segment. However, in the current cell-free assays, we observed that the NEU1–MUC1-CD interaction is direct and does not require accessory proteins, including the established NEU1-binding partners, PPCA, and β-galactosidase.

NEU1 associates with the same MUC1-CD (aa1–36) region that contains binding sites for multiple adapters and signaling proteins (14, 15). Amino acid sequences to which these MUC1-CD binding partners interact have been identified, including Tyr^20^-His-Pro-Met (PI3K and PDGFRβ), Tyr^35^-Val-Pro-Pro (phospholipase C-γ), Thr^41^-Asp-Arg-Ser (protein kinase C-δ), Asp^42^-Arg-Ser-Pro (glycogen synthase kinase-3β), Tyr^46^-Glu-Lys-Val (EGFR, c-Src), Ser^50^-Ala-Gly-Asn-Gly-Gly-Ser-Ser-Leu (β-catenin), and Tyr^60^-Thr-Asn-Pro (growth factor receptor-bound protein-2) (58, 59, 60). In other studies, MUC1-CD binding partners have been localized to the MUC1-CD without delineation of specific aa sequences. Examples of this latter group that interact predominantly or exclusively with the MUC1-CD juxtamembranous region (aa1–36) include p53, c-Met, ZAP-70, estrogen receptor α, and IkappaB kinase β, whereas proteins that associate with the MUC1-CD membrane distal region (aa37–72) include toll-like receptor (TLR)5, c-Abl, Lyn, Lck, fibroblast growth factor receptor 3, heat shock protein 70, heat shock protein 90, IkappaB kinaseγ, and NF-κB (58, 59, 60). Of note, NEU1 is reported to interact with and regulate the activity of some of these same or homologous proteins identified as MUC1-CD-binding partners, including EGFR (11), PDGFRα (61), TLR4, TLR7, TLR9 (47, 48, 49, 50, 51), and NF-κB (48, 51). With these results in mind, MUC1-CD may serve as a common signaling platform that facilitates NEU1 interaction and/or cross talk with MUC1-CD-associated signaling molecules. It is conceivable that other MUC1-CD binding partners besides PI3K might also be sterically and/or electrostatically influenced or competitively displaced from the MUC1-CD (aa1–36) region by NEU1. NEU1 association with the MUC1-CD (aa1–36) region might also disrupt the association of signaling components that interact with the membrane distal MUC1-CD (aa37–72) region. The relative binding affinities and intermolecular spatial positions of the NEU1–MUC1-CD complex *versus* other MUC1-CD-signaling protein interactions likely dictate which MUC1-CD binding partner interactions prevail.

A dense glycocalyx, which comprised numerous, terminally sialylated glycoproteins and glycolipids, covers the surface of all mammalian cells (1, 2). Although these many sialylated molecules are regulated, in part, by sialidases, only 4 mammalian sialidases (NEU1–NEU4) have been identified (3, 4, 5). NEU1 reportedly associates with and desialylates the EDs of at least 14 integral membrane glycoproteins, including MUC1, EGFR, CD5, CD31, CD36, CD44, TLR4, TLR7, TLR9, integrins β2 and β4, insulin receptor, insulin-like growth factor receptor 1, and neurotrophin (11, 33, 44, 46, 47, 48, 49, 50, 51, 52, 53, 54, 55, 56, 57). In fact, a recent proteomic analysis cataloged 71 newly identified, putative NEU1-binding partners (53). The ability of NEU1 to modify these many substrates raises the question as to how this single molecule can gain access to and recognize this diverse array of surface-expressed sialoglycoproteins. Does one unified, common pathway exist or might multiple mechanisms be operative, *e.g.*, membrane recycling from the Golgi/endoplasmic reticulum, exosomal release, and/or lysosomal exocytosis? Tyrosine phosphorylation of the COOH-terminus of NEU1 reportedly redistributes the enzyme from the lysosome to the cell surface (57). It is conceivable that a MUC1-CD-associated protein tyrosine kinase phosphorylates NEU1 to provoke its redistribution to the plasma membrane. Alternatively, NEU1 might exit the cell *via* lysosomal exocytosis. However, at least under certain experimental conditions, NEU1 is a negative regulator of lysosomal exocytosis through its desialylation of the lysosomal-associated membrane protein-1 (62). More recently, NEU1 has been detected in the cell-free culture supernatants of murine microglia cells (63). In NEU1-overexpressing COS-7 cells, the sialidase was identified in exosomes (63).

**Figure 7:**
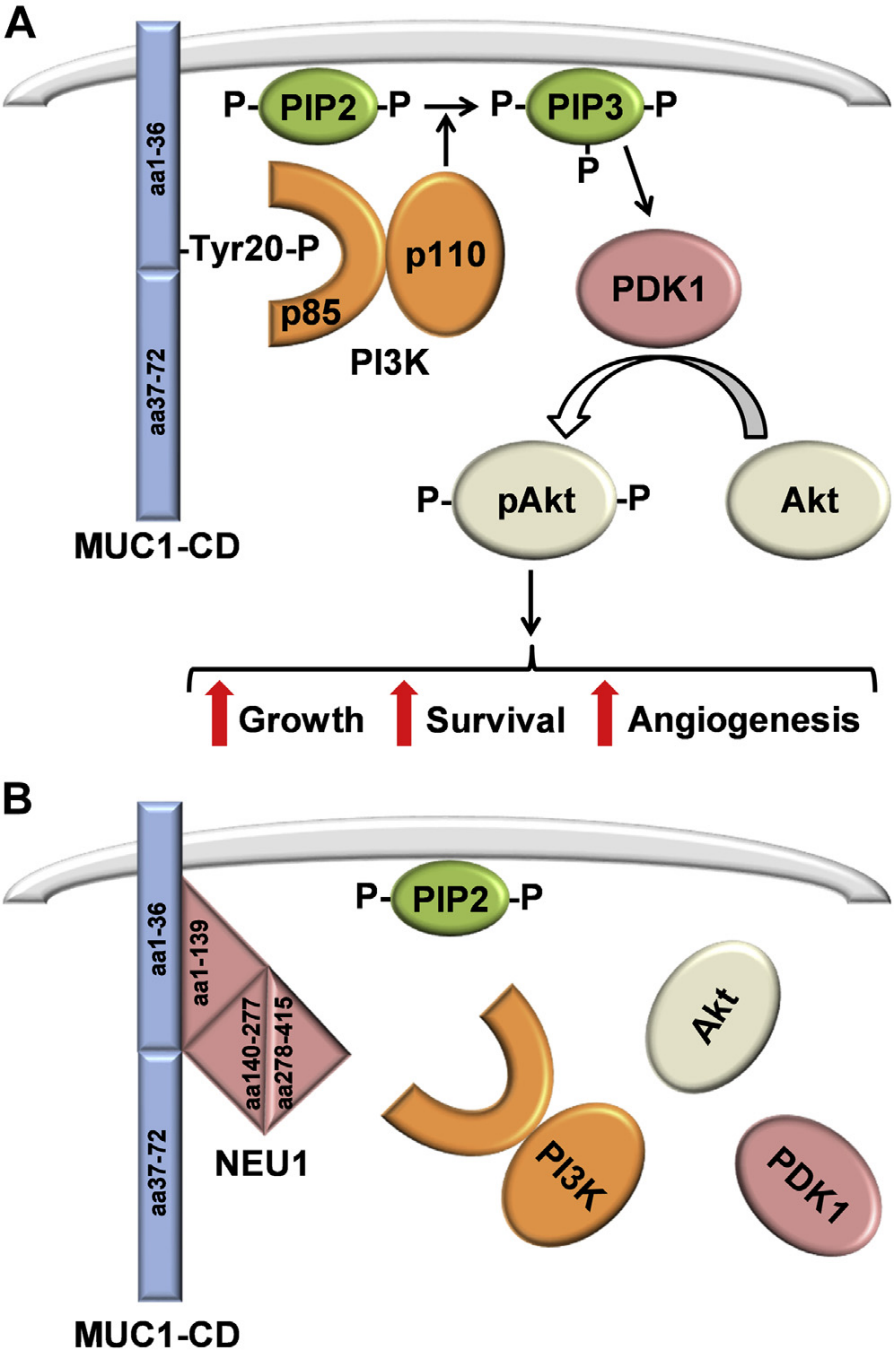
**NEU1 disrupts the MUC1-CD/PI3K/Akt pathway: A proposed model.***A*, the p85 subunit of PI3K binds to phosphorylated Tyr-20 of the MUC1-CD. The PI3K p110 catalytic subunit promotes the conversion of phosphatidylinositol-(4,5)-bisphosphate (PIP2) to phosphatidylinositol-(3,4,5)-bisphosphate (PIP3) in the plasma membrane. Phosphoinositide-dependent kinase-1 (PDK1) is recruited to membrane-bound PIP3 where it stimulates the phosphorylation of Akt, leading to downstream signaling pathways that stimulate cell growth and survival and angiogenesis. *B*, NEU1 sialidase disrupts the MUC1-CD-PI3K-Akt signaling cascade by binding to the MUC1-CD and competitively displacing its interaction with PI3K. MUC1-CD, mucin-1 cytoplasmic domain; NEU1, neuraminidase-1.

The current data support a direct protein–protein interaction between NEU1 and the MUC1-CD, whereas the MUC1-ED is a proven *in vivo* NEU1 substrate (11, 12, 13). To our surprise, we found that MUC1-CD was not required for Pa flagellin-induced, NEU1-mediated MUC1-ED desialylation. Therefore, a direct link between NEU1–MUC1-CD association and MUC1-ED desialylation could not be established. NEU1 bound to the MUC1-CD might constitute a distinct intracellular sialidase pool uninvolved in MUC1-ED desialylation but strategically positioned to influence multiple intracellular signaling events. More specifically, our demonstration that NEU1 binds to the MUC1-CD and inhibits PI3K-Akt signaling in a noncatalytic manner provides evidence to support a role for NEU1 in regulating PI3K-related events, including cell proliferation and migration, apoptosis, glucose metabolism, and angiogenesis (64) (Fig. 7). In fact, forced NEU1 overexpression in human airway epithelia dampens EGF-stimulated EGFR Tyr^1068^ autophosphorylation (11) and restrains EGFR-driven epithelial cell migration in an *in vitro* wounding assay (65). In human lung microvascular endothelia, NEU1 overexpression reduces cell migration in wounding assays (37, 66) and disrupts capillary-like tube formation on a Matrigel substrate, *i.e.*, *in vitro* angiogenesis, *via* CD31 desialylation (65, 66, 67). In a mouse model of atherosclerosis, administration of elastin-derived peptides increased atherosclerotic plaque size formation through a NEU1–PI3K pathway (68). Finally, Zhou *et al.* (69) recently reported that NEU1 overexpression in human bladder cancer cells diminished cell proliferation and enhanced apoptosis, in part, through disruption of PI3K-Akt signaling.

In previous studies, we identified a Pa-expressed flagellin–NEU1–MUC1-ED axis that releases a shed, hyperadhesive MUC1-ED decoy receptor from the airway epithelial cell surface as a protective component of the host response to Pa lung infection (11, 12, 13). In this model, Pa-derived flagellin engages the MUC1-ED to recruit the NEU1 sialidase from the lysosome to the membrane-spanning MUC1 sialoglycoprotein. NEU1 desialylates the MUC1-ED to unmask a cryptic Gly-Ser protease recognition site, thereby permitting the shedding of soluble MUC1-ED that functions as a decoy receptor to competitively inhibit Pa from establishing an invasive infection. Pa invasion of epithelial cells is mediated, in part, through the PI3K–Akt pathway (70, 71). Internalization of Pa strain PAK into Madin–Darby canine kidney or HeLa cells required activation of PI3K and Akt (70). Furthermore, Pa type IV pili mediated Pa adhesion to polarized human airway epithelial cell glycans on the apical surface, leading to activation of the PI3K-Akt signaling and bacterial invasion. However, Pa-derived flagellin engagement of heparan sulfate proteoglycans on the basolateral cell surface also activated PI3K-Akt signaling to stimulate Pa internalization (71). Together with our current data, these results suggest that NEU1, through its ability to noncatalytically disrupt the MUC1-CD–PI3K–Akt pathway, provides a host-protective mechanism to limit Pa entry into host-airway epithelia. Combined with its catalytic role in generating a hyperadhesive, shed MUC1-ED decoy receptor, NEU1 offers a bipartite level of regulation over the airway epithelial response to Pa colonization and/or infection.

## Experimental procedures

### Reagents

All reagents were from Sigma-Aldrich unless otherwise noted. The mouse anti-MUC1-ED (catalog number MA1-06503), hamster anti-MUC1-CD (catalog number MA5-11202), rabbit anti-FLAG (catalog number 740001), mouse anti-6XHis (catalog number 37-2900), horseradish peroxidase (HRP)-conjugated rabbit antimouse IgG (catalog number 31450), HRP-conjugated goat-anti-rabbit IgG (catalog number 31460), and HRP-conjugated goat antihamster IgG (catalog number PA1-28823) antibodies were from Thermo Fisher Scientific. The rabbit antibodies to NEU1 (catalog number ab233119), PPCA (catalog number ab217857), and c-Met (catalog number ab216574); and the mouse antibodies to PI3K (p85) (catalog number ab86714), p53 (catalog number ab154036), PDGFRβ (catalog number ab69506), Akt (catalog number ab108202) and phosphorylated Akt (catalog number ab105731) were from Abcam. The mouse antibodies to c-Src (catalog number 612378), EGFR (catalog number 610017), γ-catenin (catalog number 610253), and GST (catalog number 610719) were from BD Biosciences. Glutathione-agarose and Ni-NTA-agarose were from GE Healthcare Bio-Sciences. Biotinylated PNA and HRP-conjugated streptavidin were from Vector Laboratories.

### Cell culture lysates

A549-airway epithelial cells and HEK293T cells (American Type Culture Collection) were cultured in Dulbecco's modified Eagle's medium (DMEM) containing 100 units/ml penicillin, 100 μg/ml streptomycin, and 10% fetal bovine serum (Thermo Fisher Scientific). The cells were lysed on ice for 20 min in PBS, pH 7.2, 0.5% Triton X-100, 50 mM NaF, 5.0 mM EDTA, and 1.0% protease inhibitor cocktail (10 μg/ml pepstatin, 10 μg/ml leupeptin, 1.0 μg/ml aprotinin, and 0.1 mM EDTA). The lysates were centrifuged at 14,000*g* for 10 min at 4 °C, and the protein concentrations were measured by a detergent compatible Lowry assay using bovine serum albumin as standard.

### Human bronchoalveolar lavage fluid

Bronchoalveolar lavage was performed on mechanically ventilated patients undergoing diagnostic bronchoscopy, as described (12). The study was conducted in accordance with the Declaration of Helsinki, protecting patients in biomedical research and was approved by the University of Maryland Institutional Review Board (protocol number HP-00059183) with informed consent obtained from all the participants. The BALF was filtered through sterile gauze, centrifuged at 450×*g* to remove cells, and the supernatants concentrated 25-fold by passage through membrane filters (pore size, 100 kDa) in Centricon tubes (Sigma-Aldrich).

### Reciprocal NEU1-MUC1-ED and NEU1-MUC1-CD co-immunoprecipitation assays

To determine whether NEU1 binds to the MUC1-ED and/or the MUC1-CD, A549 cells were stimulated for 10 min with 10 ng/ml of Pa flagellin, washed, and lysed in PBS containing 0.5% Triton X-100. In other experiments, A549 cells were stimulated with flagellin in the presence of 100 μM of the NEU1-selective sialidase inhibitor, C9-butyl amide-2-deoxy-2,3-dehydro-N-acetylneuraminic acid (67), and lysed. The cell lysates were treated with 0.1% SDS for 20 min at room temperature to separate the noncovalently associated MUC1-ED and MUC1-CD subunits and subsequently diluted 10-fold in PBS, 1.0% Triton X-100 to allow for immunoprecipitation with anti-NEU1, anti-MUC1-ED, or anti-MUC1-CD antibodies (72). The resulting immune complexes were immobilized on protein G-agarose for 2 h at 4 °C, centrifuged, washed, boiled in SDS-PAGE sample buffer, and again centrifuged. The supernatants from NEU1 immunoprecipitates were processed for MUC1-ED or MUC1-CD immunoblotting, and the supernatants from MUC1-ED and MUC1-CD immunoprecipitates processed for NEU1 immunoblotting. To control for loading and transfer, the blots were stripped and reprobed with the immunoprecipitating antibody.

### Recombinant plasmids and proteins

The recombinant plasmids and encoded proteins used in this study are listed in Table 1. The GST fusion proteins of NEU1 (aa1–415), MUC1-CD (aa1–72), MUC1-CD (aa1–36), MUC1-CD (aa37–72), MUC1-CD (aa1–18), and MUC1-CD (aa19–36) were constructed by quantitative RT-PCR using the primers listed in Table 1, as described (73, 74). Because of the variable number of tandem repeats in the MUC1-ED, the 72-aa MUC1-CD is numbered beginning the first amino acid of MUC1-CD. The full-length NEU1 cDNA in the pBluescript II KS plasmid (6) was generously provided by Drs E. Bonten and A. d'Azzo (St Jude Children's Research Hospital) and subcloned into the *Bam*HI and *Xho*I restriction endonuclease sites of the pGEX-5X-1 plasmid (GE Healthcare Bio-Sciences) using standard procedures (75) to generate a fusion protein with an NH_2_-terminal GST tag. The MUC1-CD (aa1–72), MUC1-CD (aa1–36), MUC1-CD (aa37–72), MUC1-CD (aa1–18), and MUC1-CD (aa19–36) cDNAs were subcloned from the full-length MUC1 cDNA in the pcDNA3.1 plasmid (76) into the *Bam*HI and *Xho*I sites of pGEX-5X-1 to generate fusion proteins with NH_2_-terminal GST tags. The full-length NEU1 (aa1–415) cDNA was subcloned into the pET-22b(+) plasmid (EMD Millipore) to generate a fusion protein with a COOH-terminal 6XHis epitope tag. The recombinant MUC1-CD and NEU1 plasmids were transformed into *E. coli* BL21 competent cells, induced with 0.1 mM isopropyl 1-thio-β-D-galactoside for 4 h at 37 °C, and the expression of the resulting proteins verified on Coomassie blue-stained SDS-PAGE gels. The MUC1-ED-WT (aa1–375), and MUC1-ED-Δ1 (aa1–362), MUC1-ED-Δ2 (aa1–331), MUC1-ED-Δ3 (aa1–311), MUC1-ED-Δ4 (aa312–375), MUC1-ED-Δ5 (aa312–340), and MUC1-ED-Δ6 (aa341–375) deletion mutants were subcloned from the previously described MUC1-ED template in the pcDNA3.1 plasmid (76) into the *Xho*I and *Hin*dIII endonuclease sites of the pBAD/His-B plasmid (Thermo Fisher Scientific) to generate fusion proteins with an NH_2_-terminal 6XHis epitope tag. The recombinant MUC1-ED plasmids were transformed into *E. coli* TOP10 competent cells, induced with 0.02% L-arabinose for 4 h at 37 °C, and expression of the resulting proteins verified on Coomassie blue-stained SDS-PAGE gels.

**Table 1:** Recombinant plasmids and proteins used in this study

cDNA	Plasmid	Primer sequence	MW^a^ (kDa)
GST-MUC1-CD (aa1–72)^b^	pGEX-5X-1	F: 5′-TGTCAGTGCCGCCGAAAG R: 5′-CTACAAGTTGGCAGAAGTGG	34.9
GST-MUC1-CD (aa1–36)	pGEX-5X-1	F: 5′-TGTCAGTGCCGCCGAAAG R: 5′-CACATAGCGCCCATGGGTG	31.4
GST-MUC1-CD (aa37–72)	pGEX-5X-1	F: 5′-CCCCCTAGCAGTACCGATCG R: 5′-CTACAAGTTGGCAGAAGTGG	30.5
GST-MUC1-CD (aa1–18)	pGEX-5X-1	F: 5′-TGTCAGTGCCGCCGAAAG R: 5′-ATCCCGGGCTGGAAAGATG	29.2
GST-MUC1-CD (aa19–36)	pGEX-5X-1	F: 5′-ACCTACCATCCTATGAGCG R: 5′-CACATAGCGCCCATGGGTG	29.2
GST-NEU1 (aa1–415)	pGEX-5X-1	F: 5′-ATGACTGGGGAGCGACCCAG R: 5′-TCAGAGTGTCCCATAGACAC	72.4
GST-NEU1 (aa1–139)	pGEX-5X-1	F: 5′-ATGACTGGGGAGCGACCCAG R: 5′-AAGGTTCAGCCCATCGGG	42.1
GST-NEU1 (aa140–277)	pGEX-5X-1	F: 5′-GGGGCAGTAGTGAGCGATG R: 5′-GATGACGACTGAGCCATC	42.1
GST-NEU1 (aa278–415)	pGEX-5X-1	F: 5′-AATGCCCGAAACCAGAAC R: 5′-TCAGAGTGTCCCATAGACAC	42.1
6XHis-NEU1 (aa1–415)	pET-22b(+)	F: 5′-ATGACTGGGGAGCGACCCAG R: 5′-TCAGAGTGTCCCATAGACAC	46.3
6XHis-MUC1-ED-WT (aa1–375)	pBAD/His-B	F: 5′-CATGACACCGGGCACCCAGTC R: 5′-AGATCCTGGCCTGAACTTAAT	34.3
6XHis-MUC1-ED-Δ1 (aa1–362)	pBAD/His-B	F: 5′-CATGACACCGGGCACCCAGTC R: 5′-ACCCCCTTGTTTATAAATCTGC	32.9
6XHis-MUC1-ED-Δ2 (aa1–331)	pBAD/His-B	F: 5′-CATGACACCGGGCACCCAGTC R: 5′-AAACTGGAGGTTTGAAATGTG	29.2
6XHis-MUC1-ED-Δ3 (aa1–311)	pBAD/His-B	F: 5′-CATGACACCGGGCACCCAGTC R: 5′-GGGAGAAGTGCTGTGATTG	26.9
6XHis-MUC1-ED-Δ4 (aa312–375)	pBAD/His-B	F: 5′-CCAGTTGTCTACTGGGGTC R: 5′-AGATCCTGGCCTGAACTTAAT	7.4
6XHis-MUC1-ED-Δ5 (aa312–340)	pBAD/His-B	F: 5′-CCAGTTGTCTACTGGGGTC R: 5′-GGTGCTGGGATCTTCCAG	3.3
6XHis-MUC1-ED-Δ6 (aa341–375)	pBAD/His-B	F: 5′-CGACTACTACCAAGAGCTGC R: 5′-AGATCCTGGCCTGAACTTAAT	4.1

MW, molecular weight.

a The predicted molecular weight of GST- or 6XHis-fusion protein based on nucleotide sequence.

b Because of the variable number of tandem repeats in the MUC1-ED, the 72-aa MUC1-CD is numbered beginning the first amino acid of MUC1-CD.

### GST-NEU1 protein binding assays

To determine whether NEU1 binds to the MUC1-ED and/or the MUC1-CD, HEK293T cells were transiently transfected with recombinant pcDNA3.1 plasmids encoding for human MUC1-ED (aa1–375) or MUC1-CD (aa1–72), as described (76). At 48 h posttransfection, the cells were lysed, and equal protein aliquots of cell lysates were incubated for 3 h at 4 °C with GST-NEU1 (aa1–415) or GST alone, each immobilized on glutathione-agarose beads or with glutathione-agarose beads alone. The agarose beads were extensively washed and boiled in SDS-PAGE sample buffer, and the proteins resolved by SDS-PAGE, transferred to polyvinylidene fluoride (PVDF) membranes, and processed for MUC1-ED or MUC1-CD immunoblotting.

### 6XHis-MUC1-ED protein binding assays

A549 cells were infected with recombinant Ad-NEU1-FLAG and lysed, as described (11, 12, 13). Equal protein aliquots of Ad-NEU1-FLAG-infected cell lysates were incubated for 3 h at 4 °C with 6XHis-MUC1-ED immobilized on Ni-NTA-agarose or with Ni-NTA-agarose alone, as described (77). The agarose-bound proteins were resolved by SDS-PAGE, transferred to PVDF membranes, and processed for FLAG (NEU1) immunoblotting. In still other experiments, equal protein aliquots of Ad-NEU1-FLAG-infected cell lysates were incubated for 3 h at 4 °C with 6XHis-MUC1-ED-WT (aa1–375) or with MUC1-ED-Δ1 (aa1–362), MUC1-ED-Δ2 (aa1–331), MUC1-ED-Δ3 (aa1–311), MUC1-ED-Δ4 (aa312–375), MUC1-ED-Δ5 (aa312–340), or MUC1-ED-Δ6 (aa341–375) deletion mutant proteins, each immobilized on Ni-NTA-agarose. The agarose-bound and unbound proteins were resolved by SDS-PAGE, transferred to PVDF membranes, and processed for FLAG (NEU1) immunoblotting. In reciprocal pull-down assays, 6XHis-MUC1-ED-WT (aa1–375), 6XHis-MUC1-ED-Δ1 (aa1–362), 6XHis-MUC1-ED-Δ2 (aa1–331), 6XHis-MUC1-ED-Δ3 (aa1-311), 6XHis-MUC1-ED-Δ4 (aa312–375), 6XHis-MUC1-ED-Δ5 (aa312–340), and 6XHis-MUC1-ED-Δ6 (aa341–375) recombinant proteins were each adsorbed to Ni-NTA-agarose, eluted with PBS, pH 7.4 containing 300 mM imidazole and dialyzed against PBS, pH 7.4. Equal protein aliquots of the purified MUC1-ED-WT and deletion mutant proteins were each incubated for 3 h at 4 °C with GST-NEU1 (aa1–415) immobilized on glutathione-agarose. The agarose-bound and unbound proteins were resolved by SDS-PAGE, transferred to PVDF membranes, and processed for 6XHis (MUC1-ED) immunoblotting.

### GST-MUC1-CD protein binding assays

To identify the aa sequence(s) within the MUC1-CD that were responsible for binding to NEU1, equal protein aliquots of Ad-NEU1-FLAG-infected cell lysates were incubated for 3 h at 4 °C with GST-MUC1-CD (aa1–72), GST-MUC1-CD (aa1–36), GST-MUC1-CD (aa37–72), GST-MUC1-CD (aa1–18), GST-MUC1-CD (aa19–36), or GST alone, each immobilized on glutathione-agarose or with glutathione-agarose alone. The agarose-bound proteins resolved by SDS-PAGE and transferred to PVDF membranes. The GST-MUC1-CD (aa1–72)-, GST-MUC1-CD (aa1–36)-, GST-MUC1-CD (aa37–72)-, GST-MUC1-CD (aa1–18)-, and GST-MUC1-CD (aa19–36)-binding proteins processed for FLAG (NEU1) immunoblotting.

To establish whether the NEU1–MUC1-CD interaction was direct, the GST-NEU1-binding assay was repeated, but here GST alone or GST-MUC1-CD (aa1–72), each immobilized on glutathione agarose was incubated with NEU1 that was proteolytically cleaved from GST by Factor Xa and purified in a GST trap column, as described (73, 74), and the MUC1-CD-binding proteins processed for NEU1 immunoblotting. In other experiments, GST-MUC1-CD and GST alone, each immobilized glutathione-agarose were eluted with free glutathione and incubated with 6XHis-NEU1 coupled to Ni-NTA-agarose, and the NEU1-binding proteins processed for MUC1-CD or GST immunoblotting.

### Effect of NEU1 binding to MUC1-CD on PI3K-Akt signaling

To assess whether NEU1 overexpression might influence the interaction between the MUC1-CD and four of its established binding partners, PI3K, p53, c-Met, and PDFGRβ, A549 cells were infected with Ad-NEU1, Ad expressing a catalytically inactive NEU1 containing a Gly^68^-to-Val substitution (Ad-NEU1-G68V), or Ad expressing GFP (Ad-GFP), as described (11, 12). Equal protein aliquots of lysates of Ad-NEU1-, Ad-NEU1-G68V-, or Ad-GFP-infected cells were incubated for 3 h at 4 °C with GST-MUC1-CD (aa1–72), or GST alone, each immobilized on glutathione-agarose. The agarose-bound proteins were resolved by SDS-PAGE, transferred to PVDF membranes, and processed for PI3K (p85), p53, c-Met, or PDGFRβ immunoblotting. In still other experiments, equal protein aliquots of lysates of Ad-NEU1-, Ad-NEU1-G68V-, or Ad-GFP-infected cells were processed for pAkt immunoblotting. To control for protein loading and transfer, the immunoblots were stripped and reprobed for total Akt. The pAkt signal was quantified by densitometry and normalized to the total Akt signal in the same lane on the same stripped and reprobed blot. In all immunoblot assays described above, unmanipulated total cell lysates were included as expression and gel mobility controls for each protein of interest.

### Statistical analysis

All values were expressed as means ± S.E. Differences between means were compared using the Student's *t* test and considered significant at *p* < 0.05.

## Data availability

All data are contained within the manuscript.

## Supporting information

This article contains supporting information (67, 78).

## Conflict of interest

The authors declare that they have no conflicts of interest with the contents of this article.
