# Supplementary material for: The sialidase NEU1 directly interacts with the juxtamembranous segment of the cytoplasmic domain of mucin-1 to inhibit downstream PI3K-Akt signaling
Source: J Biol Chem. 2021 Oct 22;297(5):101337. doi: 10.1016/j.jbc.2021.101337 (PMC8591358; doi:10.1016/j.jbc.2021.101337)
Supplement: Figures S1–S3 [file mmc1.docx]

**Supplementary Information for**

The sialidase NEU1 directly interacts with the juxtamembranous segment of the cytoplasmic domain of mucin-1 to inhibit downstream PI3K-Akt signaling

Erik P. Lillehoj

[elillehoj@som.umaryland.edu](mailto:elillehoj@som.umaryland.edu)

**This file includes:**

Figs. S1-S21, the corresponding figure legends, and 2 references.

**Supporting Figures and Figure Legends**

**Figure S1. NEU1 does not associate with the MUC1-CD in BALF from patients with Pa lung infections.** (A) BALFs from patients with Pa lung infections containing the MUC1-ED but not the MUC1-CD [11] were immunopreciptated with anti-NEU1 (lanes 1-4) or anti-MUC1-ED (lanes 5-8) antibodies. The NEU1 immunoprecipitates were processed for MUC1-ED immunoblotting, and the MUC1-ED immunoprecipitates were processed for NEU1 immunoblotting. (B) To control for protein loading and transfer, the immunoblots were stripped and reprobed with the immunoprecipitating antibody. Molecular weights in kDa are indicated on the left. IP, immunoprecipitation; IB, immunoblot; IB*, immunoblot after stripping. The results are representative of 2 independent experiments.

**Figure S2. NEU1 does not associate with MUC1-ED deletion mutants.** (A) Schematic of WT MUC1-ED (aa1-375) and deletion mutants MUC1-ED-Δ1 (aa1-362), MUC1-ED-Δ2 (aa1-331), MUC1-ED-Δ3 (aa1-311), MUC1-ED-Δ4 (aa312-375), MUC1-ED-Δ5 (aa312-340), and MUC1-ED-Δ6 (aa341-375). (B, C) A549 cells were infected with Ad-NEU1-FLAG (MOI=100), cultured for 48 h, and lysed. The lysates were incubated with 6XHis-MUC1-ED-WT (aa1-375) (lane 1), 6XHis-MUC1-ED-Δ1 (aa1-362) (lane 2), 6XHis-MUC1-ED-Δ2 (aa1-331) (lane 3), 6XHis-MUC1-ED-Δ3 (aa1-311) (lane 4), 6XHis-MUC1-ED-Δ4 (aa312-375) (lane 5), 6XHis-MUC1-ED-Δ5 (aa312-340) (lane 6), or 6XHis-MUC1-ED-Δ6 (aa341-375) (lane 7), each immobilized on Ni-NTA-agarose beads, or were directly loaded on the gel (lane 8). (B) Proteins bound to the beads, and (C) proteins not retained by the beads and total lysates were processed for FLAG (NEU1) immunoblotting. (D, E) Lysates of HEK293T cells transfected for expression of 6XHis-MUC1-ED-WT (aa1-375), 6XHis-MUC1-ED-Δ1 (aa1-362), 6XHis-MUC1-ED-Δ2 (aa1-331), 6XHis-MUC1-ED-Δ3 (aa1-311), 6XHis-MUC1-ED-Δ4 (aa312-375), 6XHis-MUC1-ED-Δ5 (aa312-340), or 6XHis-MUC1-ED-Δ6 (aa341-375) were each incubated with Ni-NTA-agarose beads and then eluted with imidazole. The purified WT MUC1-ED and deletion mutant proteins (lanes 1-4, 6-8), or lysates of HEK-293T transfected for MUC1-CD expression (lanes 5, 9), were incubated with GST-NEU1 immobilized on glutathione-agarose beads. (D) Proteins bound to the beads, (E) proteins not retained by the beads, and HEK-293T cell lysates were processed for 6XHis (MUC1-ED) (lanes 1-4,6-8) or MUC1-CD (lanes 5, 9) immunoblotting. (B-E) Molecular weights in kDa are indicated on the left. PD, pull-down; IB, immunoblot. The results are representative of 2 independent experiments.

**Figure S3. MUC1-CD and NEU1 constructs.** (A) Secondary structure prediction algorithms (PSIPRED, http://bioinf.cs.ucl.ac.uk/psipred/) (1) identify three distinct regions of the MUC1-CD. The recombinant MUC1-CD constructs used in this study each contain one or more of these elements (the initial β-strand and/or α-helices 1 and 2) as depicted. (B) Based on our previous homology model of NEU1 (2), the recombinant NEU1 constructs used in this study include non-overlapping distinct regions of the catalytic domain, none of which include the complete active site.

**References**

1. Buchan, D. W. A., and Jones, D. T. (2019) The PSIPRED protein analysis workbench: 20 years on. *Nucleic Acids Res.* **47(W1)**, W402-W407.

2. Hyun, S. W., Liu, A., Liu, Z., Cross, A. S., Verceles, A. C., Magesh, S., Kommagalla, Y., Kona, C., Ando, H., Luzina, I. G., Atamas, S. P., Piepenbrink, K. H., Sundberg, E. J., Guang, W., Ishida, H., Lillehoj, E. P., and Goldblum S. E. (2016) The NEU1-selective sialidase inhibitor, C9-butyl-amide-DANA, blocks sialidase activity and NEU1-mediated bioactivities in human lung *in vitro* and murine lung *in vivo*. *Glycobiology* **26,** 834-849.

**Figure S4. Full-length immunoblots for original Figure 2B.** A549 cells were lysed and the lysates incubated with glutathione-agarose beads alone (lane 1) or GST-NEU1 immobilized on glutathione-agarose beads (lane 2), or were directly loaded on the gel (lane 3). Proteins bound to the beads and total lysates were processed for PPCA immunoblotting. The arrow indicates the bands of interest shown in original Figure 2B. Molecular weights in kDa are indicated on the left. PD, pull-down; IB, immunoblot.

**Figure S5. Full-length immunoblots for original Figure 2C.** HEK293T cells were transfected for MUC1-ED (lanes 1-4) or MUC1-CD (lanes 5-8) expression, cultured for 48 h, and lysed. The lysates were incubated with beads alone (lanes 1, 5), GST (lanes 2, 6), or the validated GST-NEU1 (lanes 3, 7), each immobilized on the beads, or were directly loaded on the gel (lanes 4, 8). Proteins bound to the beads and total lysates were processed for MUC1-ED (lanes 1-4) or MUC1-CD (lanes 5-8) immunoblotting. The arrows indicate the bands of interest shown in original Figure 2C. Molecular weights in kDa are indicated on the left. PD, pull-down; IB, immunoblot.

**Figure S6. Full-length immunoblots for original Figure 2E.** A549 cell lysates were incubated with GST or GST-MUC1-CD (aa1-72), each immobilized on glutathione-agarose beads, or were directly loaded on the gel. Proteins bound to the beads and total lysates were processed for c-Src (lanes 1-3), EGFR (lanes 4-6), or γ-catenin (lanes 7-9) immunoblotting. The arrows indicate the bands of interest shown in original Figure 2E. Molecular weights in kDa are indicated on the left. PD, pull-down; IB, immunoblot.

**Figure S7. Full-length immunoblots for original Figure 2F.** A549 cells were infected with Ad-NEU1-FLAG at a multiplicity of infection (m.o.i.) = 100, cultured for 48 h, and lysed. Lysates were incubated with GST or the validated GST-MUC1-CD (aa1-72), each immobilized on glutathione-agarose beads, or were directly loaded on the gel. Proteins bound to the beads and total lysates were processed for FLAG (NEU1) immunoblotting. The arrow indicates the bands of interest shown in original Figure 2F. Molecular weights in kDa are indicated on the left. PD, pull-down; IB, immunoblot.

**Figure S8. Full-length immunoblots for original Figure 2H.** Purified Pa-derived flagellin was incubated with Ni-NTA-agarose beads alone (lane 1) or 6XHis-MUC1-ED immobilized on Ni-NTA-beads (lane 2), or was directly loaded on the gel (lane 3). Proteins bound to the beads and the loaded flagellin gel mobility control were processed for flagellin immunoblotting. The arrow indicates the bands of interest shown in original Figure 2H. Molecular weights in kDa are indicated on the left. PD, pull-down; IB, immunoblot.

**Figure S9. Full-length immunoblots for original Figure 2I.** A549 cells were infected with Ad-NEU1-FLAG (m.o.i. = 100), incubated for 48 h, and lysed. The lysates were incubated with Ni-NTA-agarose beads alone (lane 1) or the validated 6XHis-MUC1-ED immobilized on Ni-NTA-beads (lane 2), or were directly loaded on the gel (lane 3). Proteins bound to the beads and total lysates were processed for FLAG (NEU1) immunoblotting. The arrow indicates the bands of interest shown in original Figure 2I. Molecular weights in kDa are indicated on the left. PD, pull-down; IB, immunoblot.

**Figure S10. Full-length immunoblots for original Figure 3B.** A549 cells were infected with Ad-NEU1-FLAG (m.o.i. = 100), cultured for 48 h, and lysed. Lysates were incubated with GST-MUC1-CD (aa1-36) or GST-MUC1-CD (aa37-72) each immobilized on glutathione-agarose beads, or were directly loaded on the gel. Proteins bound to the beads and total lysates were processed for FLAG (NEU1) immunoblotting. The arrow indicates the bands of interest shown in original Figure 3B. Molecular weights in kDa are indicated on the left. PD, pull-down; IB, immunoblot.

**Figure S11. Full-length immunoblots for original Figure 3C.** A549 cells were infected with Ad-NEU1-FLAG (m.o.i. = 100), cultured for 48 h, and lysed. Lysates were incubated with GST-MUC1-CD (aa1-18) or GST-MUC1-CD (aa19-36) each immobilized on glutathione-agarose beads, or were directly loaded on the gel. Proteins bound to the beads and total lysates were processed for FLAG (NEU1) immunoblotting. The arrow indicates the bands of interest shown in original Figure 3C. Molecular weights in kDa are indicated on the left. PD, pull-down; IB, immunoblot.

**Figure S12. Full-length immunoblots for original Figure 3D.** A549 cells were infected with Ad-NEU1-FLAG (m.o.i. = 100), cultured for 48 h, and lysed. Lysates were incubated with GST or GST-MUC1-CD (aa1-72) each immobilized on glutathione-agarose beads, after which the beads were incubated with Factor Xa to proteolytically release the bound proteins, and the released proteins purified on a GST trap column. The eluted proteins and total lysates were processed for FLAG (NEU1) immunoblotting. The arrow indicates the bands of interest shown in original Figure 3D. Molecular weights in kDa are indicated on the left. PD, pull-down; IB, immunoblot.

**Figure S13. Full-length immunoblots for original Figure 3E.** GST (lane 1) and GST-MUC1-CD (lane 2), each immobilized on glutathione-agarose beads, were eluted with free glutathione and incubated with 6XHis-NEU1 coupled to Ni-NTA-agarose beads. The 6XHis-NEU1-binding proteins, and purified GST-MUC1-CD (lane 3), were processed for MUC1-CD immunoblotting. The arrow indicates the bands of interest shown in original Figure 3E. Molecular weights in kDa are indicated on the left. PD, pull-down; IB, immunoblot.

**Figure S14. Full-length immunoblots for original Figure 3F.** GST (lane 1) and GST-MUC1-CD (lane 2), each immobilized on glutathione-agarose beads, were eluted with free glutathione and incubated with 6XHis-NEU1 coupled to Ni-NTA-agarose beads. The 6XHis-NEU1-binding proteins, and purified GST-MUC1-CD (lane 3), were processed for GST immunoblotting. The arrow indicates the bands of interest shown in original Figure 3F. Molecular weights in kDa are indicated on the left. PD, pull-down; IB, immunoblot.

**Figure S15. Full-length immunoblots for original Figure 4B.** A549 cell lysates were incubated with GST, GST-NEU1 (aa1-139), GST-NEU1 (aa140-277), or GST-NEU1 (aa278-415), each immobilized on glutathione-agarose beads, or were directly loaded on the gel. Proteins bound to the beads and total lysates were processed for PPCA immunoblotting. The arrow indicates the bands of interest shown in original Figure 4B. Molecular weights in kDa are indicated on the left. PD, pull-down; IB, immunoblot.

**Figure S16. Full-length immunoblots for original Figure 5.** HEK293T cells were transfected with the empty pcDNA plasmid (lanes 1, 2), or with plasmids encoding the full-length (FL) MUC1 (lanes 3, 4), MUC1-ED (lanes 5, 6), or MUC1-ED plus MUC1-CD (lanes 7, 8), and cultured for 48 h. The cells were incubated for 30 min with 10 ng/ml of Pa-expressed flagellin or medium alone, and lysed. The lysates were immuno-precipitated with anti-MUC1-ED antibody and the immunoprecipitates processed for PNA lectin blotting (upper panel). To control for loading and transfer, the blots were stripped and reprobed for MUC1-ED (middle panel) and MUC1-CD (lower panel). The arrows indicate the bands of interest shown in original Figure 5. Molecular weights in kDa are indicated on the left. IP, immuno-precipitation; IB, immunoblot; IB*, immunoblot after stripping.

**Figure S17. Full-length immunoblots for original Figure 6A.** A549 cells were infected with Ad-GFP, Ad-NEU1, or Ad-NEU1-G68V (m.o.i. = 100), cultured for 48 h, and lysed. The lysates were incubated with GST (lane 1) or GST-MUC1-CD (aa1-72) (lanes 2-4), each immobilized on glutathione-agarose beads, or were directly loaded on the gel (lane 5). Proteins bound to the beads and total lysates were processed for PI3K immunoblotting. The arrow indicates the bands of interest shown in original Figure 6A. Molecular weight in kDa is indicated on the left. PD, pull down; IB, immunoblot.

**Figure S18. Full-length immunoblots for original Figure 6B.** A549 cells were infected with Ad-GFP, or Ad-NEU1 (m.o.i. = 100), cultured for 48 h, and lysed. The lysates were incubated with GST (lane 1) or GST-MUC1-CD (aa1-72) (lanes 2, 3), each immobilized on glutathione-agarose beads, or were directly loaded on the gel (lane 4). Proteins bound to the beads and total lysates were processed for p53 immunoblotting. The arrow indicates the bands of interest shown in original Figure 6B. Molecular weight in kDa is indicated on the left. PD, pull down; IB, immunoblot.

**Figure S19. Full-length immunoblots for original Figure 6C.** A549 cells were infected with Ad-GFP, or Ad-NEU1 (m.o.i. = 100), cultured for 48 h, and lysed. The lysates were incubated with GST (lane 1) or GST-MUC1-CD (aa1-72) (lanes 2, 3), each immobilized on glutathione-agarose beads, or were directly loaded on the gel (lane 4). Proteins bound to the beads and total lysates were processed for c-Met immunoblotting. The arrow indicates the bands of interest shown in original Figure 6C. Molecular weight in kDa is indicated on the left. PD, pull down; IB, immunoblot.

**Figure S20. Full-length immunoblots for original Figure 6D.** A549 cells were infected with Ad-GFP, or Ad-NEU1 (m.o.i. = 100), cultured for 48 h, and lysed. The lysates were incubated with GST (lane 1) or GST-MUC1-CD (aa1-72) (lanes 2, 3), each immobilized on glutathione-agarose beads, or were directly loaded on the gel (lane 4). Proteins bound to the beads and total lysates were processed for PDGFRβ immunoblotting. The arrow indicates the bands of interest shown in original Figure 6D. Molecular weight in kDa is indicated on the left. PD, pull down; IB, immunoblot.

**Figure S21. Full-length immunoblots for original Figure 6E.** A549 cells were infected with Ad-GFP, Ad-NEU1, or Ad-NEU1-G68V (m.o.i. = 100), cultured for 48 h, and lysed. The lysates were processed for pAkt immunoblotting (upper panel). To control for protein loading and transfer, the immunoblot was stripped and reprobed for total Akt (lower panel). The arrows indicate the bands of interest shown in original Figure 6E. Molecular weight in kDa is indicated on the left. PD, pull down; IB, immunoblot; IB*, immunoblot after stripping.
